# Structure Characterization, Immunological Activity, and Mechanism of a Polysaccharide From the Rhizome of *Menispermum dauricum* DC

**DOI:** 10.3389/fnut.2022.922569

**Published:** 2022-06-15

**Authors:** Pei Yang, Juan Jin, Yan Ma, Fengshan Wang, Yaying Li, Baoguo Duan, Yongqing Zhang, Yuhong Liu

**Affiliations:** ^1^School of Pharmaceutical Sciences, Collaborative Innovation Center for Quality Control and Construction of the Whole Industrial Chain of Traditional Chinese Medicine, Shandong University of Traditional Chinese Medicine, Jinan, China; ^2^National Medical Products Administration Key Laboratory for Quality Research and Evaluation of Carbohydrate-Based Medicine, Jinan, China; ^3^Experimental Center, Shandong University of Traditional Chinese Medicine, Jinan, China; ^4^Sishui Siheyuan Culture and Tourism Development Company, Ltd., Sisui, China

**Keywords:** *Menispermum dauricum* DC, polysaccharide, structure characterization, immunological activity, TLR4

## Abstract

The purpose of this study was to investigate the structural characterization and immunological activity *in vitro* and *in vivo* of a polysaccharide from the rhizome of *Menispermum dauricum*. A new polysaccharide named MDP was isolated from the rhizome of *Menispermum dauricum* by hot water extraction, ethanol precipitation, anion-exchange, and gel-filtration chromatography. MDP was homogeneous and had a molecular weight of 6.16 ×10^3^ Da, and it was an α-D-glucan containing a (1 → 6)-linked backbone, with a glucosyl residue at the C-3 position along the main chain. MDP exhibited immunological activity *in vitro*, which could significantly promote the proliferation and phagocytosis of RAW264.7 cells and the release of TNF-α and IL-6 factors. For immunological activity *in vivo*. MDP could significantly increase the thymus and spleen indices, enhance the macrophage function, increase the level of cytokine (IL-6 and TNF-α) and immunoglobulin IgM in the serum and regulate T lymphocyte subsets. Furthermore, MDP elevated the expression of the critical nodes in the TLR4-MyD88 signaling pathways *in vivo*. These results support the concept that MDP may exhibit immunological activity through TLR4-MyD88 signaling pathway in *vivo*.

## Introduction

The immune system is an essential defense system which can defend against foreign invasion. It has the capacity to cooperate with other body systems to sustain the stability and physiological balance of the body (1, 2). Immunosuppression is the inhibition of the immune response, which can bring about a variety of diseases such as urinary tract infection, meningitis, and sepsis (1, 2). Therefore, maintaining the normal state of the immune system to reduce immunosuppression is significant in preventing the occurrence of various diseases.

The traditional treatment of immunosuppression has so far been mainly chemical drugs. The immunopotentiators commonly used in clinical practice currently include levamisole, isoprinosine, interferon, interleukin, BCG, etc. Among them, levamisole and isoprinosine are chemically synthesized drugs; interferon, interleukin and BCG are originated from microorganisms (3). However, these drugs are generally accompanied by many side effects, such as nausea, vomiting, abdominal pain and other gastrointestinal reactions; some drugs may lead to anaphylactic shock and even death, take BCG, for example (4). Due to the drawbacks of pharmaceutical therapy, the interest of researchers in the active ingredients of natural origin had remarkably increased throughout the past decades, especially for polysaccharide components of various traditional Asian medicines. Many pharmacological effects of polysaccharides have been recently discovered, including liver protection, resistance to oxidation and aging, and anticancer properties. Polysaccharides obtained from various traditional medicinal plants also had been proven to exert profound effects on the immune system *in vivo* and *in vitro* through their capacity to modulate immune function, including cytokine/chemokine production, reactive oxygen species (ROS) production, and cell proliferation. It has promising possibilities as an immunomodulator having no significant side effects (5).

The rhizome of *Menispermum dauricum DC* (Menispermaceae), called Bei Dou Gen in Chinese, is a traditional Chinese medicinal herb that has been used widely in clinical practice for treating rheumatic arthralgia, dysentery, colitis, and sore throats (6). Alkaloids are the main chemical components of the rhizome of *M. dauricum* and possess various bioactivities, including antiarrhythmic, antitumor and cardiovascular effects (7–9). Moreover, the injection of total alkaloids has been applied clinically for a long time to treat chronic tracheitis, throat sores and arthralgia (10). Nevertheless, the polysaccharides from the rhizome of *M. dauricum* have received little attention. Only a few researchers reported its preparation, antitumor (11, 12), and anti-mutagenic (13) activities. Unfortunately, there was no research on the structure and the immunological activity of the polysaccharide of the rhizome of *M. dauricum*, which greatly limits its further development and utilization. Thus, an in-depth study of the immunological activity and structural characterization of polysaccharides from the rhizome of *M. dauricum* requires thorough studies.

In the present research, isolation of the novel polysaccharide (MDP) was conducted from the rhizome of *M. dauricum* and its structural characterization was performed. Furthermore, the immunological effects and potential mechanism of action of MDP were carried out.

## Materials and Methods

### Materials and Chemicals

Shandong Baiweitang Chinese Herbal Pieces Co., Ltd. (Jinan, Shandong Province, China) supplied the rhizome of *M. dauricum* (No. 181201). The voucher sample of rhizome of *M. dauricum* was deposited in the School of Pharmaceutical Sciences of Shandong University of Traditional Chinese Medicine, Jinan, China.

Chemicals: Yuanye Biological Co., Ltd. (Shanghai, China) supplied the DEAE-cellulose-52. GE Healthcare Life Sciences (Piscataway, NJ, USA) supplied Sephadex G-50 and Sephacryl S-100. Guoyao Group Co., Ltd. (Beijing, China) supplied galactose and glucose. Macklin Biochemical Technology Co., Ltd. (Shanghai, China) supplied arabinose and xylose. The purchase of DMEM was achieved from GIBCO (USA) with supplementing of 1% streptomycin, 10% FBS, and 1% penicillin. Sigma-Aldrich (St. Louis, MO, USA) supplied LPS. Enzyme-linked Biotechnology Co., Ltd. (Shanghai, China) supplied ELISA kits, which were used in the NO, TNF-α, IL-6, and IgM tests. Injectable levamisole (LMS) was purchased from Yuanye Biological Co., Ltd. (Shanghai, China). Injectable cyclophosphamide (CTX) was purchased from Jiangsu Hengrui Medicine Co. (Lianyungang, Jiangsu, China). Antibodies against MyD88, NFκB, and JNK were purchased from ABclonal (Wuhan, China) and other antibodies were obtained from Cell Signaling Technology (Beverly, MA, USA).

### Extraction and Purification of Polysaccharide

The extraction of crude polysaccharides from the rhizome of *M. dauricum* was conducted with distilled water every 3 h at a temperature of 87°C at a ratio of 20:1 (w/w). The aqueous extract was concentrated under vacuum after three rounds of extraction. Subsequently, four times the volume of ethanol was added for the purpose of precipitating the polysaccharide, and the mixture was allowed to stand at 4°C overnight (14). The precipitate was collected and deproteinized by means of the TCA-n-butanol method (15) and then subjected to freeze-drying so as to yield a crude polysaccharide fraction (CMDP).

The CMDP was purified with the DEAE-52 cellulose column (5.5 ×30 cm) and eluted using distilled water as well as 0.1 M, 0.2 M, 0.3 M, and 0.5 M NaCl. The fraction eluted using 0.2 M NaCl was found to have the highest polysaccharides yield. As a result, it was collected and dialyzed to remove NaCl and then further purified by Sephacryl S-100 column (1.75 ×66 cm) and Sephadex G-50 column (1.75 ×66 cm) eluted with deionized water. The solution obtained after elution was then collected based on the phenol-sulfuric acid approach, and the major fraction was collected and lyophilized for the purpose of acquiring a white purified polysaccharide (MDP).

### Determination of Total Carbohydrate and Protein

The determination of the contents of total carbohydrates and proteins was performed by performing a phenol-sulfuric acid test (16) as well as a Folin–phenol test (17), respectively.

### Analysis of the Molecular Weight

The estimations for the homogeneity and average molecular weight of MDP were performed with the aid of the Agilent 1200 system (Agilent Technologies, Palo Alto, CA, USA) utilizing the high-performance gel permeation chromatography (HPGPC) coupled with PL aquagel-OH MIXED-M gel column (7.5 ×300 mm, Agilent, Palo Alto, CA, USA) as well as refractive index detector (RID, Agilent Technologies, Palo Alto, CA, USA). The column was subjected to an elution at a flow rate of 1 mL/min with the use of a 0.1 mol/L NaNO_3_. Dextrans having different molecular weights (Sigma, USA) were utilized as the standard for molecular weight determination (18).

### Structure Characterization

#### Analysis of the Monosaccharide Composition

An analysis of the MDP monosaccharide composition was conducted by means of thin-layer chromatography (TLC) and GC-MS (Agilent Technologies, USA) coupled with an HP-5 capillary column (30 m ×250 μm i.d., 0.25 μm film thickness). MDP (10 mg) was added to 6 mL of 2 mol/L trifluoroacetic acid (TFA) and hydrolyzed at 110°C for 3 h. Through concentrating under reduced pressure, the mixture was classified into two parts after removing TFA. TLC was used to analyze one part of the hydrolysate to determine whether the sample was hydrolyzed completely and contained uronic acid. Monosaccharide standards and hydrolyzed MDP were acetylated through the addition of acetic anhydride and pyridine, after which analysis was performed by GC-MS. The temperature program was 170°C for 3 min, 170–178°C at a rate of 0.5°C/min for 3 min, and then increased to 210°C for 5 min at a rate of 2°C/min.

#### Methylation Analysis

Methylation of polysaccharides was carried out in accordance with Need's method with some adjustments (19). After methylation, hydrolysis, reduction, and acetylation, which are the basic steps of methylation, the polysaccharide samples were subjected to GC-MS analysis. The dried MDP (10 mg) was dissolved with the use of 3 mL of dimethyl sulfoxide and stirred at room temperature until the polysaccharide sample was dissolved entirely. Sodium hydroxide (60 mg) was added and ground into a fine powder, and then the mixture was stirred for 1 h at room temperature. After the reaction, 2.5 mL of CH_3_I was gradually dropped into the sample under a nitrogen environment, and the reaction was continued at 20°C for 1 h while avoiding light. For the purpose of terminating the reaction, 2 mL of distilled water was then added. Three extractions of the methylated polysaccharides were performed with 5 ml of chloroform, and the chloroform extract was collected and then extracted with deionized water 4 times to remove water-soluble impurities in the chloroform extract. This methylation procedure was carried out 4 times, and confirmation of the full methylation was established based on the absence of hydroxyl peaks in the IR spectrum.

Depolymerization of the dried permethylated product was performed at 100°C with 90% HCOOH for 6 h and further hydrolyzed for 3 h with the use of 2 M TFA at 110°C. Reducting of the residues was conducted with NaBH_4_, followed by acetylation with the use of the acetic anhydride. Lastly, redissolving of the methylated alditol acetates were achieved in CHCl_3_, followed by GC-MS analysis. The GC temperature program was 170°C for 3 min, 170–178°C for 3 min at a rate of 0.5°C/min, and increased to 210°C for 5 min at a rate of 2°C/min. These alditol derivatives were obtained by GC-MS database and published literature combined with the relative retention times on GC-MS, and the assessment of the molar ratios was completed according to the response factors and the peak areas.

#### Periodate Oxidation and Smith Degradation

The reported approach was used in treating the MDP sample (20). In short, MDP was dissolved in 15 mmol/L sodium periodate and kept in darkness at room temperature. Monitoring of NaIO_4_ consumption was performed with the aid of a UV-2550 spectrophotometer every 12 h at 223 nm until the absorption value observed became stable. After the completion of the oxidation reaction, excess NaIO_4_ was removed through the addition of ethylene glycol. Determination for the production of formic acid was performed by titration with 0.01 M NaOH. The reaction solution was then dialyzed for 48 h (MW cutoff: 500 Da) and the addition of 50 mg of NaBH_4_ was done to reduce and dialyze again. Next, 2 M TFA was added to hydrolyze the polysaccharide solution. After complete hydrolysis, methanol was added under a reduced pressure to remove the acid by evaporation. Lastly, by performing GC-MS analysis, we investigated the acetylated product.

#### Analysis for FT-IR and UV Spectroscopy

MDP was ground with the use of KBr powder, after which it was pressed into pellets. Then, FT-IR analysis was conducted on Fourier transform infrared (FT-IR, PerkinElmer Co., Ltd., USA) instrument in the 4,000–400 cm^−1^ region. We recorded the UV spectra of MDP in a 200–400 nm wavelength range with the aid of a UV-2800 UV-visible spectrophotometer (Shimadzu Inc., Japan).

#### NMR Spectroscopy Analysis

A total of 20 mg MDP fraction that was already dried was kept over P_2_O_5_ under vacuum for several days, followed by dissolving in 0.5 mL of D_2_O. The ^1^H-^13^C HSQC, HMBC, ^1^H NMR, ^13^C NMR, and ^1^H-^1^H COSY spectra were observed using a Bruker AV-600 spectrometer (Germany) at 28°C.

### Immunological Activity *in vitro*

#### Cell Culture

We acquired RAW264.7 cells from Beina Chuanglian Biotechnology Co., Ltd. (Beijing, China). The cells were subjected to incubation in DMEM supplemented with penicillin (100 units/mL), 10% FBS, and streptomycin sulfate (100 μg/mL) at a temperature of 37°C in a humidified 5% CO_2_ incubator atmosphere.

#### Cell Viability Test

The impact of MDP at different concentrations on RAW264.7 cell viability was explored by performing the MTT assay *in vitro*. In short, RAW264.7 cells were seeded at a density of 1 ×10^4^ cells/well into 96-well microplates. After the cells were subjected to incubation at 37°C for 24 h with 5% CO_2_, then incubated for 24 h with MDP samples (0, 10, 50, 100, 200, and 400 μg/mL) or LPS (1 μg/mL, positive control) additionally. After incubation, an addition of 20 μL of the MTT solution (5 mg/mL) was added to each well and then incubated again at 37°C for an additional 4 h in the medium. Afterward, each well was subjected to treatment with 150 μL of DMSO for the purpose of dissolving formazan crystals after careful aspiration of the medium. Finally, we utilized a microplate reader (BioTek Instruments Inc., Winooski, VT, USA) with the purpose of detecting the absorbance at 570 nm.


$$\text{Cell viability }\%\;=\;(OD_{\text{ treatment group}})/(OD_{\text{ control group}})\times 100\%.$$

#### NO, TNF-α, and IL-6 Measurement

After incubation of RAW264.7 cells for 24 h with polysaccharide samples as shown above, determination of the expression levels of NO, TNF-α, and IL-6 in the culture supernatants was performed with the aid of the enzyme-linked immunosorbent assay (ELISA) kits (ML BIO Biotechnology, Shanghai, China). Assays were conducted following the instructions provided by the manufacturer, and cytokine concentrations were computed based on the standard curves.

#### Phagocytic Activity Determination

An analysis of the phagocytic activity was performed through flow cytometry. RAW264.7 macrophages were cultured in an incubator at a density of 1 ×10^5^ cells/well in a 24-well plate for 24 h. Subsequently, cells were subjected to incubation for 24 h with MDP (50, 100, 200, and 400 μg/mL) or LPS (1 μg/mL), after which the medium was changed with 1 mL PBS comprising 100 μL FITC-dextran (1 mg/mL). The plate was then allowed to be incubated in an incubator for an additional 30 min. Afterward, phagocytosis was halted through the addition of 2 mL of ice-cold PBS, after which cold PBS was utilized for the purpose of washing the cells 3 times (21). Flow cytometry was used in analyzing the phagocytic activity (Beckman, Sanjose, CA, USA) using Flow Jo software.

#### Measurement of Reactive Oxygen Species (ROS)

RAW 264.7 cells (1 ×10^5^ cells/mL) were subjected to seeding in a 6-well flat-bottom plate and incubation for 24 h. Each well was cultured with the aid of several concentrations of MDP solutions (50, 100, 200, and 400 μg/mL). The positive control group was treated with 50 μg/mL Rosup provided in the kit for 30 min. Next, the cells were exposed for an additional 30 min to DCFH-DA (10 μM), after which they were washed 3 times with PBS. Finally, flow cytometry was utilized in measuring fluorescence.

### Immunological Activity *in vivo*

#### Animal Treatment and Experimental Design

SPF Male BALB/c mice (18–22 g) were procured from Weitonglihua Laboratory Animal Technology Co., Ltd. (Beijing, China). The animals were provided with water and mouse chow *ad libitum*, and were housed in a rodent facility at 22 ± 1°C with a 12 h light-dark cycle for acclimatization. After 5 days environmental adaptation period, mice were randomly divided into five groups: the control group, CTX model group, the positive group (CTX+LMS), the low and high doses of MDP groups (CTX+MDP-L and CTX+MDP-H). MDP groups were treated with MDP (100, 200 mg/kg, i.g.) once daily on a continuous basis for 14 days. All experiments used 12 mice per group. The positive group was given LMS at a dose of 10 mg/kg (i.p.) at the same frequency. While only saline was given to the control and CTX groups. On the 10–12th days, CTX (80 mg/kg, i.p.) was given to all the groups with the exception of the control group to induce the state of immunosuppression.

#### Phagocytosis of Mononuclear

Mice were given a tail vein injection of diluted India ink (100 μL/10 g). Blood samples were collected from the retroorbital vein at 2 min (t_1_) and 10 min (t_2_), and 20 μL of blood samples were mixed with 2 mL of 0.1% sodium carbonate solution. The absorbance was measured by UV-Vis spectrophotometer at 675 nm, where OD_1_ was the absorbance at t_1_ and OD_2_ was the t_2_. The phagocytic index was calculated as the following formula:


(1) $$\begin{array}{l} \text{K}=({\text{lgOD}}_{1}{\text{−lgOD}}_{2})\text{/}({\text{t}}_{2}{\text{−t}}_{1})\\ \text{Phagocytic index α}=\sqrt[{3}]{\text{K}}\times \text{A/}(\text{B+ C}) \end{array}$$

Where A is the body weight, B is the liver weight and C is the spleen weight.

#### Spleen and Thymus Indices

Twenty-four hours after the last dose, mice were weighed and sacrificed. The spleen and thymus were removed and weighed. The spleen and thymus indices were calculated according to the following formula: thymus or spleen index (mg/g) = (weight of thymus or spleen/body weight).

#### Measurements of IgM, IL-6, and TNF-α in Serum

Serum levels of IgM, IL-6, and TNF-α were determined by colorimetry. The colorimetry was read with an enzymatic reader using the mouse ELISA kit according to the instructions.

#### Histochemical Examinations of Spleen

The Hematoxylin and eosin (HE) staining of the spleen tissues were performed to assess the histopathological condition and photographed under the microscope.

#### Immunohistochemistry of Spleen

Positioning of CD4 and CD8 was visualized by immunohistochemical staining as previously described (22). Histological sections (4 μm) were prepared from formalin-fixed, paraffin-embedded tissues. After deparaffinization and rehydration for antigen retrieval, slides were heated in 10 mM citrate buffer (pH 6.0) in a microwave oven and cooled to room temperature.

#### Western Blot Assay

Spleen tissue stored at −80°C was centrifuged (12,000 *g*, 20 min, 4°C) after homogenization in an ice bath with lysis buffer for 5 min. BCA assay was used to determine the protein content. The proteins denatured were separated with loading onto 10% SDS-PAGE by electrophoresis and transferred to PVDF membranes. 10% skim milk (made in TBS that contains 0.1% Tween 20) was utilized to block the membrane at room temperature for 4 h. The membranes were incubated with primary antibodies against TLR4 (1:1000), MyD88 (1:1500), p-NFκB (1:1000), NFκB (1:1500), p-JNK (1:2000), JNK (1:1500), p-ERK (1:1500), ERK (1:1500), p-P38 (1:1000), P38 (1:750) according to the manufacturer's instructions. After being washed 3 times with TBST, the membranes were incubated with the secondary antibody (1:5000) at room temperature for 1 h. The signal was detected with an ECL chemiluminescence detection kit (Millipore, Massachusetts, USA) and quantified by Amersham Imager 600 Chemiluminescence imaging system (GE, Boston, USA).

### Statistical Analysis

All data obtained were expressed as mean ± standard deviation (SD). One-way method of variance (ANOVA) was used to determine the statistical significance of different groups. The SPSS 20.0 software was used for all statistical analyses, ^*^P < 0.05 was regarded as statistically significant.

## Results and Discussion

### Extraction and Purification of MDP

CMDP was acquired from the rhizome of *M. dauricum* with a yield of 3.00 ± 0.14% (w/w), after a number of processing steps, which include hot water extraction, ethanol precipitation, and deproteinization by the TCA-n-butanol method. CMDP was purified through DEAE-cellulose with 0.2 M NaCl solution as the eluent and then further purified by Sephacryl S-100 and Sephadex G-50 chromatography with deionized water for the purpose of obtaining a purified polysaccharide (MDP), the yield of MDP was 0.72 ± 0.05%. The results showed that MDP is predominantly composed of polysaccharides (95.32%) with almost no protein (0.38%).

### Physicochemical Properties of MDP

**Figure 1:**
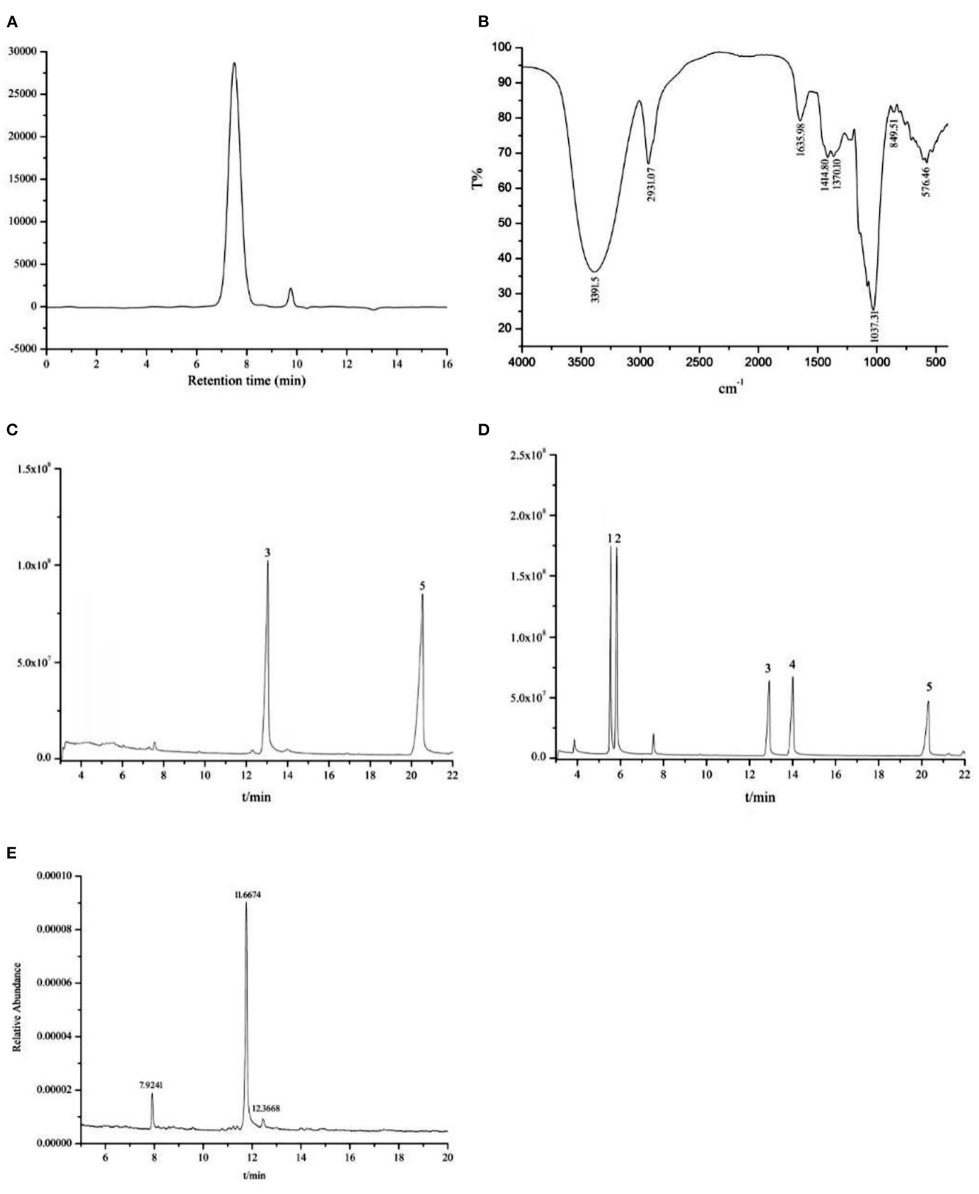
The physicochemical properties and structure characterization of MDP. **(A)** HPGPC chromatogram of MDP; **(B)** FT-IR of MDP. **(C)** GC-MS study of the monosaccharide composition of MDP; **(D)** GC-MS analysis of mixed standards. (1) Ara; (2) Xyl; (3) Glc; (4) Gal; (5) internal standard; **(E)** GC-MS analysis of the MDP methylation product.

The appearance of MDP was a white powder. Monitoring UV absorption at 280 or 260 nm revealed that there were hardly any proteins or nucleic acids. The HPGPC elution profile of MDP (Figure 1A) displayed a single symmetrical and narrow peak, suggesting that it was a homogeneous polysaccharide. According to the calibration curve of the dextran standard (y = −0.7982x + 9.753, *r* = 0.9992) obtained by Agilent GPC software, the molecular weight of MDP was ~6.16 × 10^3^ Da. The FT-IR spectrum of MDP (Figure 1B) revealed that the absorption was highly significant at 3,391.5 cm^−1^, which attributed to the angular vibration and stretching vibration of the O-H linkage of the polysaccharide. The signal at 2,931.07 cm^−1^ contributed to the stretching vibration of C-H in the sugar ring. The presence of α-configuration glycosidic bonds in MDP was confirmed by a characteristic absorption at 849.51 cm^−1^.

### Structural Characterization of MDP

TLC (Supplementary Figure 1) and GC-MS analysis (Figures 1C,D) suggested that MDP did not contain uronic acid and consisted only of glucose.

The completely methylated MDP product was hydrolyzed with acid, transformed into alditol acetate, and subjected to a GC–MS analysis (Figure 1E). Three main peaks were observed with retention times of 7.9421, 11.6674, and 12.3668, and the three kinds of derivatives were identified as 1,5-di-O-acetyl-2,3,4,6-tetra-O-methyl-glucitol, 1,5,6-tri-O-acetyl-2,3,4-tri-O-methyl-glucitol, and 1,3,5,6-tetra-O-acetyl-2,4-di-O-methyl-glucitol. The results revealed that MDP was composed of (1 → 6)-linked, (1 → 3, 6)-linked, and terminal glucosyl residues in a molar ratio of 31.27:1.00:2.24 (Table 1), indicating that MDP has a backbone consisting of (1 → 6)-linked glucosyl residues, with a single glucose branch at the C-3 position and one terminal glucosyl residue at the non-reducing end together with the main chain.

**Table 1:** GC–MS data of alditol acetate derivatives from the methylated product of MDP.

**Methylated sugars (as alditol acetates)**	**Linkage type**	**Molar** **ratio**	**Mass fragments (m/z)**
2,3,4,6-O-Me_4_-Glc	Glcp-(1 →	2.24	43; 71; 87; 101; 117; 129; 145; 161; 205
2,3,4-O-Me_3_-Glc	→ 6)-Glcp-(1 →	31.27	43; 87; 113; 117; 129; 161; 173; 189; 233
2,4-O-Me_2_-Glc	→ 3,6)-Glcp-(1 →	1.00	43; 87; 99; 101; 117; 129; 189; 233

The periodate oxidation concerning MDP led to a consumption of 2.04 mol of periodate and output of a formic acid of 1.03 mol per sugar residue, suggesting the presence of 1 → or 1 → 6 linkages. The degradation products following Smith degradation of the oxidized product were subjected to GC-MS analysis. Glycerol was mainly found, suggesting a large amount of 1 →, 1 → 6, or 1 → 2 linkages. The periodate that was consumed was about twice the formic acid obtained, suggesting that there was no 1 → 2 linkage. These findings were in good agreement with multiple studies regarding MDP methylation.

**Figure 2:**
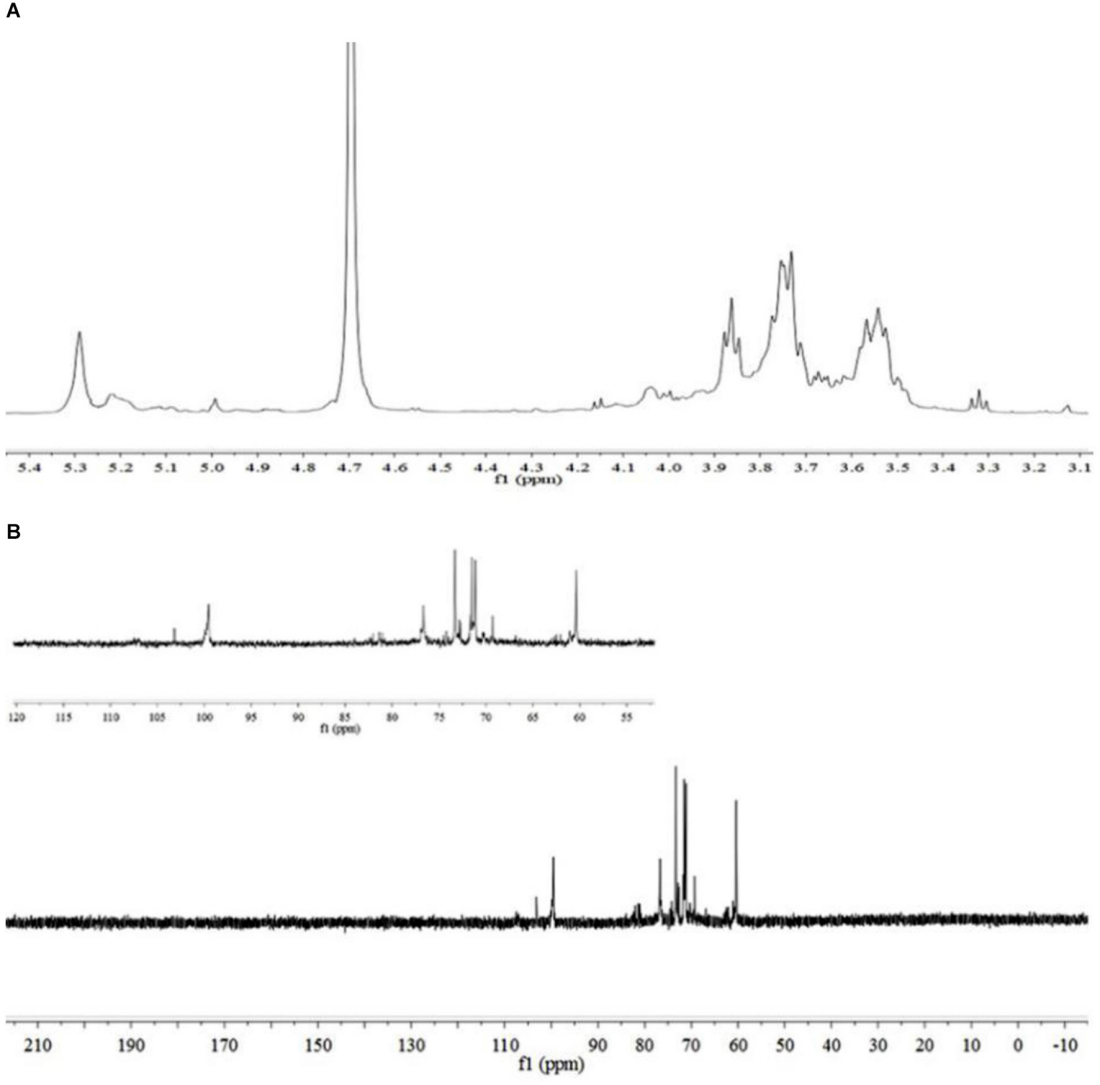
NMR spectra of MDP. **(A)** ^1^H NMR spectrum of MDP and **(B)** ^13^C NMR spectrum of MDP.

**Figure 3:**
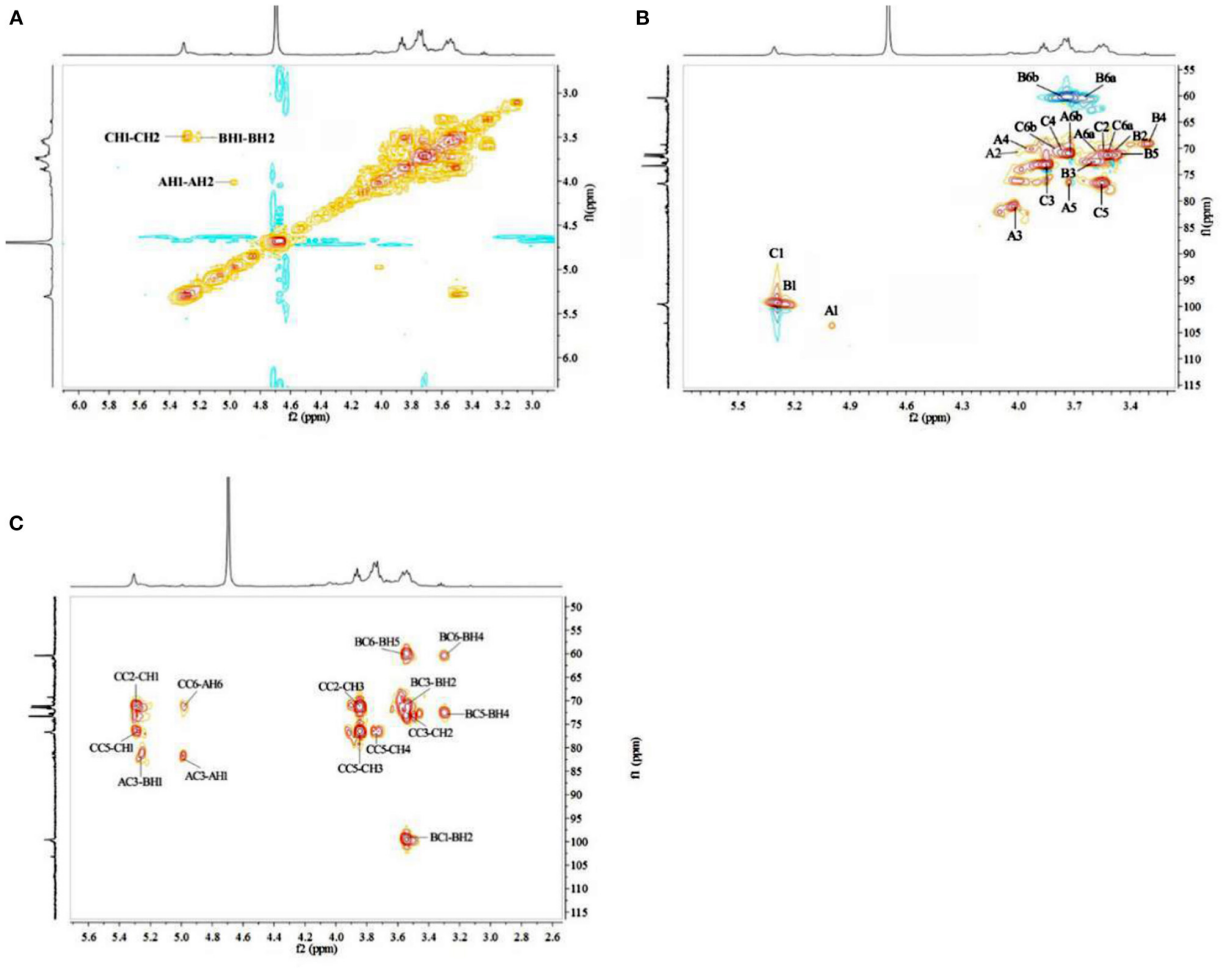
2D-NMR spectra of MDP. **(A)** ^1^H–^1^H COSY spectrum of MDP, **(B)** ^1^H–^13^C HSQC spectrum of MDP, and **(C)** ^1^H–^13^C HMBC spectrum of MDP.

**Figure 4:**
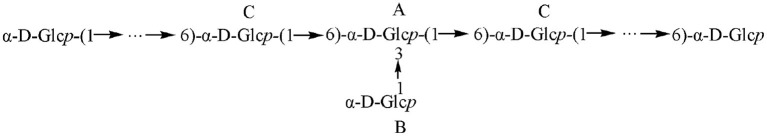
Schematic of the structure of MDP.

The sugar residues were assigned by applying 1D (^1^H NMR and ^13^C NMR) and 2D (^1^H–^1^H COSY, ^1^H–^13^C HSQC, and HMBC) NMR spectra. As most sets of signals overlap, there was a challenge in the identification of non-anomeric proton signals in the MDP ^1^H NMR spectrum (Figure 2A). However, there was a presence of three anomeric proton signals in the ^1^H-^1^H COSY correlations (Figure 3A), confirming the presence of three anomeric protons at δ 4.97, 5.23, and 5.29, which were allocated to H-1 of (1 → 3,6)-linked (A), (1 →)-linked (B), and (1 → 6)-linked (C) glucosyl residues (Figure 4), respectively. The sugar residues A, B and C were all α-configuration units according to the chemical shifts for the anomeric protons as well as the published literature comparison (23).

Three anomeric signals at δ 99.52, 99.81, and 103.25 were included in the ^13^C NMR spectrum (Figure 2B) and they were assigned to C-1 of (1 → 6)-linked (C), (1 →)-linked (B), and (1 → 3,6)-linked (A) glucosyl residues. The C-3 signal of (1 → 3,6)-linked glucosyl residues and the C-6 of (1 → 3,6)-linked, (1 → 6)-linked glucosyl residues underwent a downfield shift caused by the glycosylation effect. The signal at δ 81.09 was assigned to C-3 of (1 → 3,6)-linked glucosyl residues, and the signals at δ 71.40 and 71.47 were assigned to O-substituted C-6 of (1 → 3,6)-linked and (1 → 6)-linked glucosyl residues, respectively.

The identification of three groups with related signals was achieved from the COSY spectrum (Figure 3A): H-1/H-2 at δ 4.97/4.00, H-2/H-3 at δ 4.00/4.03, H-3/H-4 at δ 4.03/3.92, H-4/H-5 at δ 3.92/3.72, H-5/H-6a, H-6b at δ 3.72/3.52, 3.73; H-1/H-2 at δ 5.23/3.53, H-2/H-3 at δ 3.53/3.60, H-3/H-4 at δ 3.60/3.31, H-4/H-5 at δ 3.31/3.52, H-5/H-6a, H-6b at δ 3.52/3.66, 3.77; H-1/H-2 at δ 5.29/3.52, H-2/H-3 at δ 3.52/3.85, H-3/H-4 at δ 3.85/3.75, H-4/H-5 at δ 3.75/3.55, and H-5/H-6a, H-6b at δ 3.55/3.52, 3.73.

The ^1^H-^13^C HMQC (Figure 3B) spectrum of MDP suggested data on the relationship of ^13^C and its linked ^1^H: H-1/C-1 at δ 4.97/103.25, H-2/C-2 at δ 4.00/70.25, H-3/C-3 at δ 4.03/81.09, H-4/C-4 at δ 3.92/70.11, H-5/C-5 at δ 3.72/76.37 and H-6a, H-6b/C-6 at δ 3.52, 3.73/71.40 for (1 → 3,6)-linked glucosyl residue; H-1/C-1 at δ 5.23/99.81, H-2/C-2 at δ 3.53/70.28, H-3/C-3 at δ 3.60/73.19, H-4/C-4 at δ 3.31/69.20, H-5/C-5 at δ 3.52/73.12 and H-6a, H-6b/C-6 at δ 3.66, 3.77/60.36 for (1 →)-linked glucosyl residue; H-1/C-1 at δ 5.29/99.52, H-2/C-2 at δ 3.52/71.47, H-3/C-3 at δ 3.85/73.55, H-4/C-4 at δ 3.75/71.00, H-5/C-5 at δ 3.55/76.86, and H-6a, H-6b/C-6 at δ 3.52, 3.73/71.47 for (1 → 6)-linked glucosyl residue.

To deduce glycosidic bonds between sugar residues in MDP, long-range proton-carbon correlations were determined using ^1^H-^13^C HMBC (Figure 3C). In the anomeric region of the HMBC spectrum, associations from H-1 (δ 4.97) of the (1 → 3,6)-linked glucosyl residue to C-6 (δ 71.47) of the (1 → 6)-linked glucosyl residue and from H-1 (δ 5.23) of the (1 →)-linked glucosyl residue to C-3 (δ 81.09) of the (1 → 3,6)-linked glucosyl residue were found. MDP had a (1 → 6)-linked backbone, and a (1 →)-linked glucosyl residue was connected to the C-3 of the main chain according to this finding. The entire structural features of MDP were obtained from the above results, and the proton and carbon chemical shifts of different glucose residues are displayed in Table 2.

**Table 2:** ^1^H and ^13^C NMR chemical shifts of polysaccharide MDP in D_2_O.

**Linkage type**	**H-1/C-1**	**H-2/C-2**	**H-3/C-3**	**H-4/C-4**	**H-5/C-5**	**H-6/C-6**
→ 3,6)-Glcp-(1 →	4.97/103.25	4.00/70.25	4.03/81.09	3.92/70.11	3.72/76.37	3.52,3.73/71.40
Glcp-(1 →	5.23/99.81	3.53/70.28	3.60/73.19	3.31/69.20	3.52/73.12	3.66,3.77/60.36
→ 6)-Glcp-(1 →	5.29/99.52	3.52/71.47	3.85/73.55	3.75/71.00	3.55/76.86	3.52,3.73/71.47

On the above basis of the results, it can be concluded as follows: MDP is an α-D-glucan that has a (1 → 6)-linked backbone, with a single glucose branch at the C-3 position in the main chain. The predicted structure is shown in Figure 4.

### Immunological Activities of MDP *in vitro*

#### Effect of MDP on RAW264.7 Cell Viability

**Figure 5:**
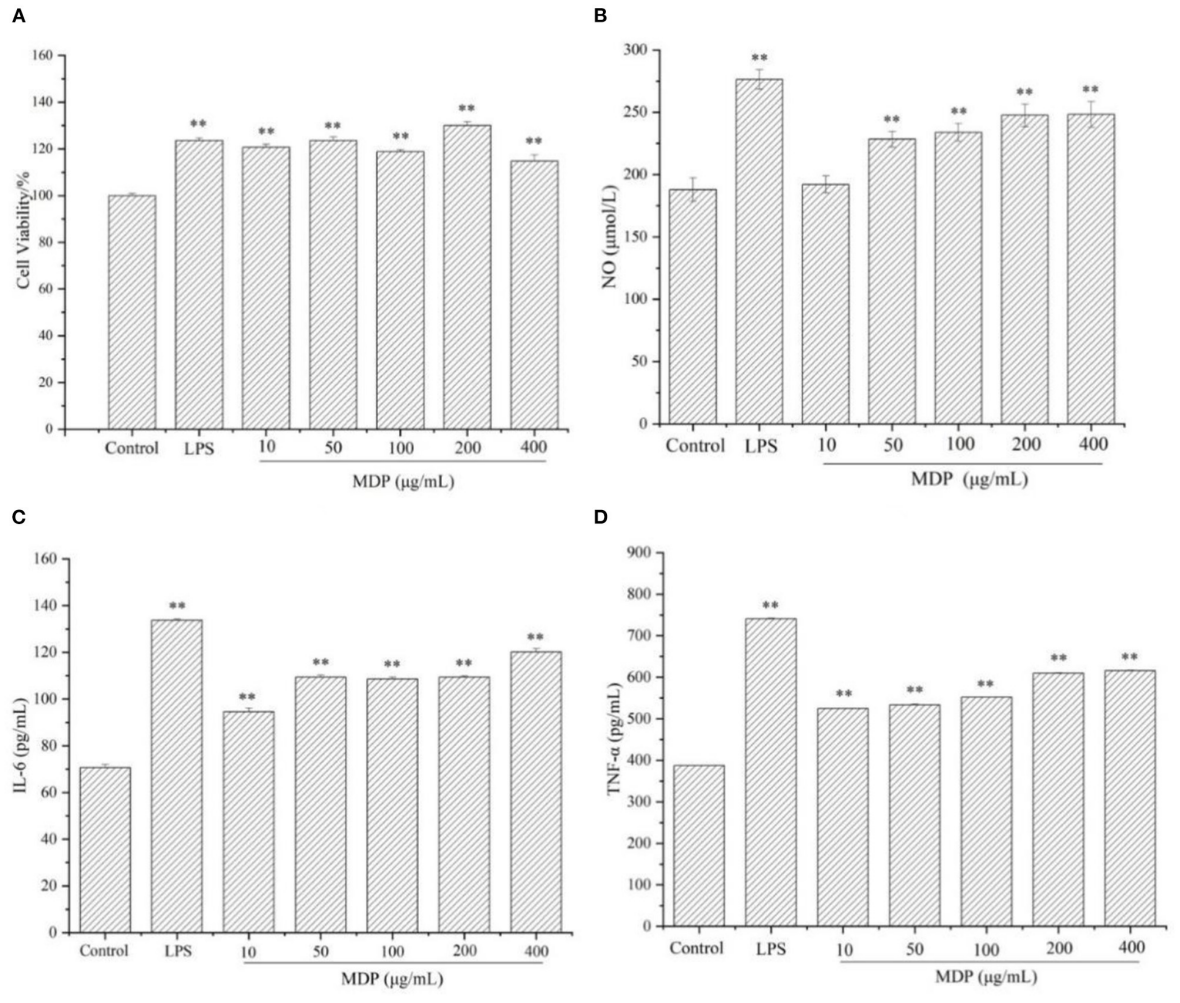
The immunological activities of MDP *in vitro*. **(A)** Effects of MDP on the viability of RAW264.7 cells. **(B)** Effect of MDP on the secretion of NO in RAW264.7 cells. **(C)** Effect of MDP on the secretion of the cytokine IL-6 in RAW264.7 cells. **(D)** Effect of MDP on the secretion of the cytokine TNF-α in RAW264.7 cells. The values are presented as the mean ± SD, *n* = 3. **P < 0.01 vs. control group.

In the innate immune system, Macrophages are the key cells. Furthermore, they are the primary cells underlying host inflammatory and other immune processes. A number of polysaccharides have been proven to promote activity and proliferation of RAW264.7 cells (24). Therefore, we assessed the influence of MDP on RAW264.7 cell viability, and the findings were presented in Figure 5A. The cell viability levels of different groups were 120.66, 123.64, 118.76, 130.02, and 114.73%, respectively, at dosages of 10, 50, 100, 200, and 400 μg/mL, (*P* > 0.05), which showed that MDP could remarkably enhance RAW264.7 proliferation.

#### Effect of MDP on the Secretion of NO, TNF-α, and IL-6 in RAW264.7 Cells

Macrophages are the most crucial immune defense-related cells in the body. After activation, they can generate a variety of cytokines and chemokines. The release of a series of biological factors (NO, TNF-α, IL-6, and etc.) is a fundamental mechanism of immunomodulators (25). As a type of essential molecule generated by macrophages, NO exerts a crucial role in apoptosis regulation and host defense against tumor cells and pathogens. Moreover, NO might also enhance the phagocytosis as well as the lysis of macrophages. As a result, the capacity of releasing NO by macrophages indicates the impacts of polysaccharides on immune function. The primary active molecules in organisms, including IL-6 and TNF-α, play crucial functions in the process of inflammation, cancer, and immune diseases (26). When the host is invaded by exogenous pathogen threats, activated macrophages generate IL-6 and TNF-α to mediate the immune system (27). The results were displayed in Figure 5. After MDP administration, there was a remarkable elevation in the expression of NO (Figure 5B), IL-6 (Figure 5C), and TNF-α (Figure 5D) levels. These results suggested that MDP has significant immunomodulatory activities *via* the mechanism of increasing the release of NO and cytokines (IL-6, TNF-α) in RAW264.7 cells.

#### Effect of MDP on the Phagocytic Activity of RAW264.7 Cells

**Figure 6:**
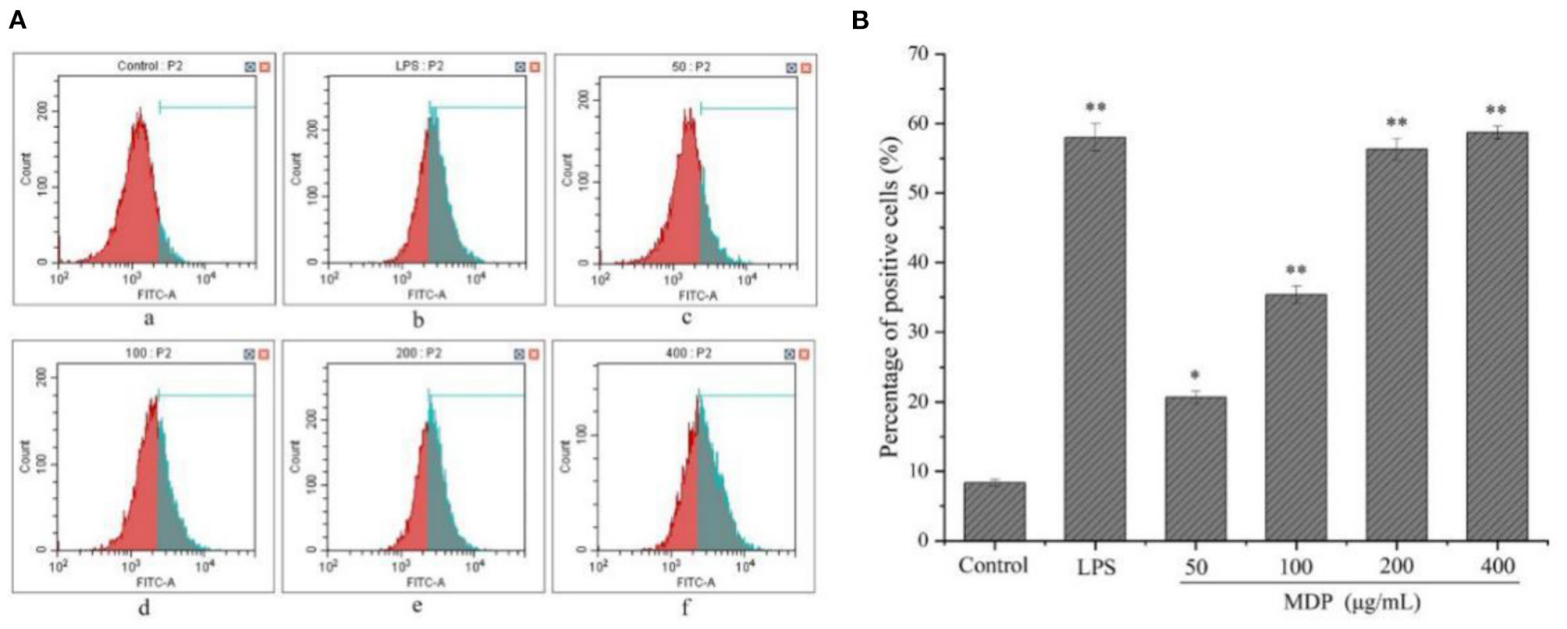
Impacts exerted by MDP on the RAW264.7 cells' phagocytic activity. **(A)** Assessment for the phagocytic activity was performed through the use of the flow cytometry (a) Control group; (b) 1 μg/mL LPS; (c) 50 μg/mL MDP; (d) 100 μg/mL MDP; (e) 200 μg/mL MDP; (f) 400 μg/mL MDP. **(B)** Quantitative analysis of the RAW264.7 cells' phagocytic activity. The values are reported as the mean ± SD, *n* = 3. *P < 0.05 or **P < 0.01 vs. control group.

Macrophages have the ability to swallow big foreign particles and multiple organelles or macromolecules in the cells, thus exerting a critical role in resisting infection and maintaining normal physiological functions. The phagocytic ability of FITC-dextran in RAW264.7 cells was performed with the aid of flow cytometry (Figure 6). In comparison to the control group, 50–400 μg/mL MDP treatment improved the RAW264.7 cells' phagocytic capacity in a dose-dependent way, the percentages of positive cells in MDP-induced RAW264.7 cells (50, 100, 200, and 400 μg/mL) were 20.65, 35.33, 56.28, and 58.69%, respectively. These results revealed that MDP has significant immunomodulatory activity by moderately improving the phagocytic ability of the large molecules in RAW264.7 cells.

#### Effect of MDP on the Production of ROS in RAW264.7 Cells

**Figure 7:**
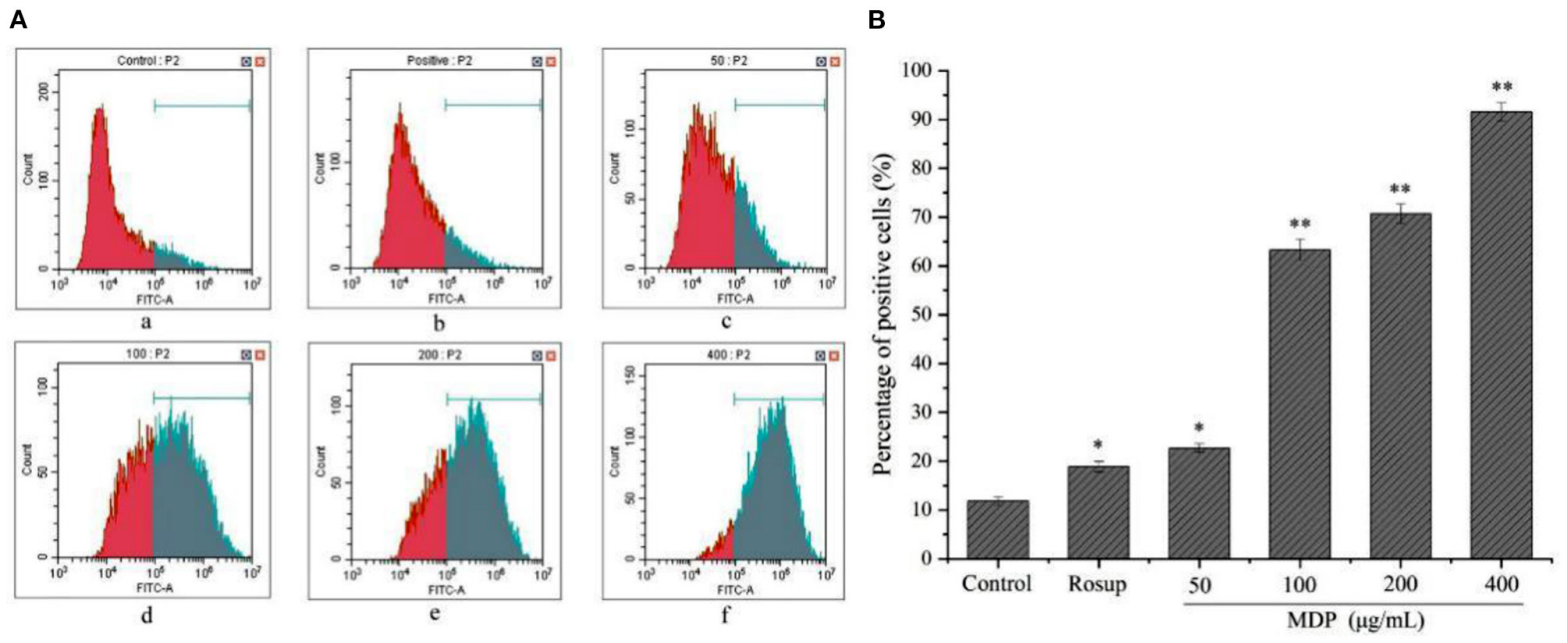
Effect of MDP on the production of ROS in RAW264.7 cells. **(A)** The production of ROS was examined by flow cytometry (a) Control group; (b) 50 μg/mL Rosup; (c) 50 μg/mL MDP; (d) 100 μg/mL MDP; (e) 200 μg/mL MDP; (f) 400 μg/mL MDP. **(B)** Quantitative analysis of the ROS production in RAW264.7 cells. The values are reported as the mean ± SD, *n* = 3. *P < 0.05 or **P < 0.01 vs. control group.

ROS plays a role in the production of multiple inflammatory factors or capacity enhancement of cells to phagocytose, eliminating bacteria as well as other foreign materials (28). Thus, the concentration of intracellular ROS is a viable biomarker for indicating the immuno-stimulating impact of samples in RAW264.7 cells. As demonstrated in Figure 7, the intensity of DCF fluorescence enhanced gradually with the increase of the dosage of MDP. In other words, the generation amount of ROS was elevated significantly in a dose-dependent way in comparison to the control group, indicating that MDP might improve the immune ability by stimulating the production of ROS.

### Immunological Activities of MDP *in vivo*

#### Effect of MDP on the Phagocytosis of Mononuclear Macrophages

**Figure 8:**
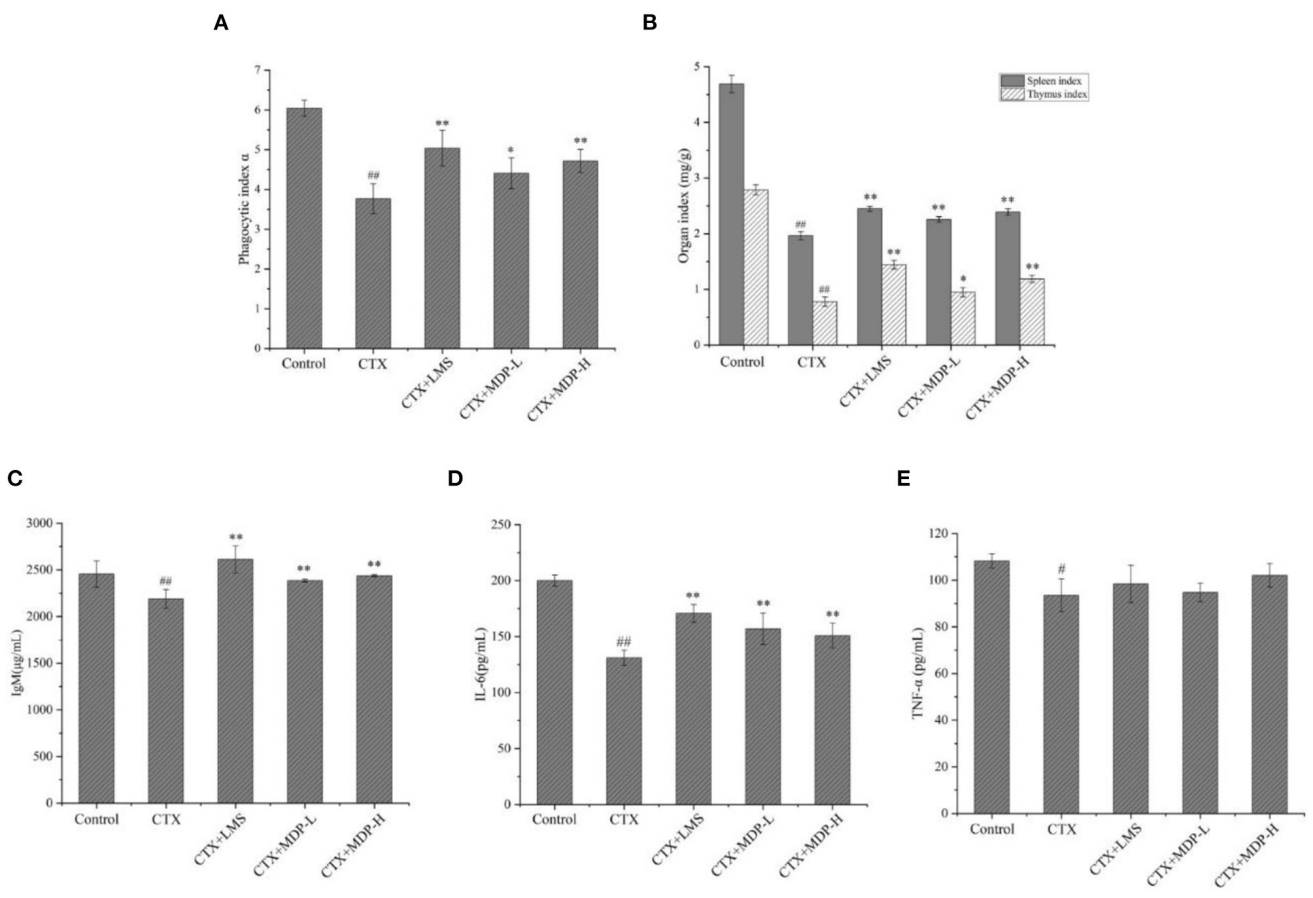
The immunological activities of MDP in *vivo*. **(A)** Effect of MDP on the phagocytic index α. **(B)** Effect of MDP on the spleen and thymus indices in mice induced by CTX. **(C)** Effect of MDP on the secretion of IgM in serum of mice induced by CTX. **(D)** Effect of MDP on the secretion of IL-6 in serum of mice induced by CTX. **(E)** Effect of MDP on the secretion of TNF-α in serum of mice induced by CTX. Values are given as means ± S.D [*n* = 3 of **(A,C–E)** and *n* = 12 of **(B)**]. ^#^*P* < 0.05, ^##^*P* < 0.01 vs. control group; *P < 0.05, **P < 0.01 vs. CTX group.

The carbon clearance tests can reflect the monocytes' phagocytic function. The removal rate of carbon particles was found to be related to the enhancement of phagocytosis (1). The phagocytic ability of mononuclear macrophages is usually denoted by the phagocytic index α. As shown in Figure 8A, it was observed that the phagocytic index α was markedly decreased in the model group compared with the control group (*P* < 0.01). MDP were effective in increasing the phagocytic index α in CTX-treated mice in a dose-dependent manner, and the phagocytic activities were nearly reinstated to the control levels at a dose of 200 mg/kg, demonstrating that MDP capable of improving the macrophage function in CTX-induced mice.

#### Effect of MDP on the Bodyweight, Spleen, and Thymus Indices

Body weight is an indicator of the growth status of mice, while thymus and spleen are the major organs of immunity, so they can be a reflection of the immunity functions of mice induced by CTX. The effect of MDP on the bodyweight of mice was presented in Table 3. CTX was found to cause a marked reduction in bodyweight to just 17.92 g compared to the control value of 22.42 g, which indicated that the immunosuppressive modeling was built successfully. Bodyweight was significantly restored when mice were treated with MDP, indicating that MDP could alleviate the weight loss caused by CTX. The spleen and thymus indices were shown in Figure 8B. Compared with the control group, CTX-treatment mice had much lower thymus and spleen indices, reflecting the worse immune activity. The thymic and splenic indices were ameliorated in LMS than in model groups, and MDP also increased the splenic and thymic indexes in CTX-induced BALB/c mice, which suggested that MDP could reverse the immune organ atrophy induced by CTX.

**Table 3:** Effect of MDP on the body weight in mice induced by CTX.

**Groups**	**Final bodyweight**	**Increase of**
		**bodyweight**
Control	22.42 ± 0.47	3.18 ± 0.44
CTX	17.92 ± 1.03^##^	−1.63 ± 0.74
CTX+LMS	19.93 ± 0.38**	−1.00 ± 0.41
CTX+MDP-L	18.94 ± 0.51*	−0.83 ± 0.31
CTX+MDP-H	19.10 ± 0.41*	−0.12 ± 0.36

*Values are given as means ± S.D (n = 12).*

*^##^P <0.01 vs. control group.*

*P <0.05, **P <0.01 vs. CTX group.

#### Effect of MDP on Cytokines and Immunoglobulin in Serum

Immuneglobulin (Ig) and cytokines are both engaged in immune response and regulation. Thus, we measured the releases of IgM, IL-6 and TNF-α in serum by ELISA according to the manufacturer's protocols. The results were shown in Figures 8C–E, the serum levels of IgM, IL-6 and TNF-α in the model group reduced significantly compared to the control group (*P* < 0.01). However, the addition of MDP resulted in an increase of IgM and IL-6 in immunosuppressive mice. MDP also increased the levels of TNF-α, but there was no significant difference. These results suggested that MDP could enhance the cytokine and immunoglobulin levels in serum of mice dramatically.

#### Effect of MDP on the Histological Morphology of Spleen

**Figure 9:**
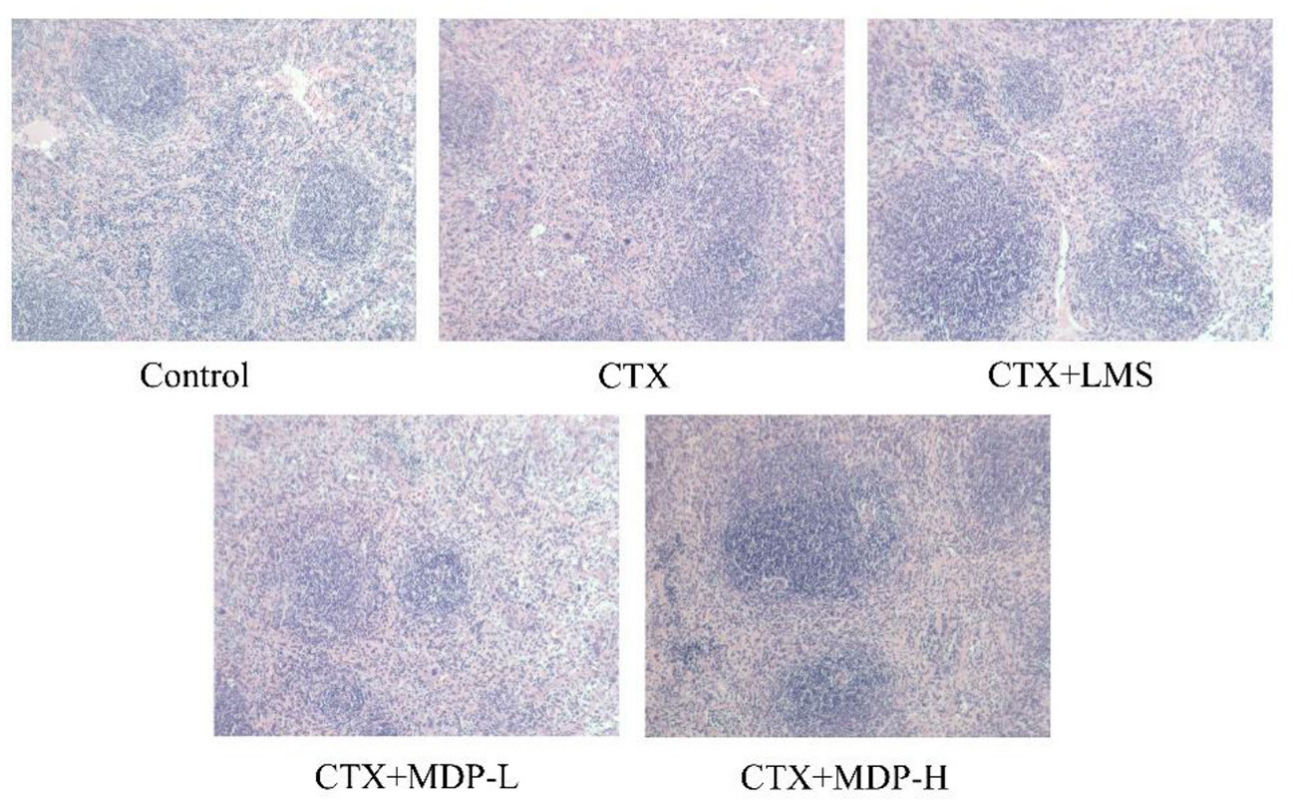
Effect of MDP on the histological morphology of spleen in mice induced by CTX.

As shown in Figure 9, clear demarcation between the white and the red pulp was observed in the spleen of the control group, and the spleen cells were dense and arranged in good order with a clear nucleus. In contrast, in the CTX group, no visible demarcation was observed between the spleen white and red pulp. What' more, CTX group displayed the small splenic corpuscle with irregular shape and discrete lymphocyte arrangement. After the intervention of MDP, the area of splenic corpuscle increased, and the dividing line became obvious. This result showed that MDP could repair the damage of CTX on the spleen through protecting the splenic corpuscle and recovering lymphocyte quantity.

#### Effect of MDP on the CD4^+^ and CD8^+^ T Lymphocytes of Spleen

**Figure 10:**
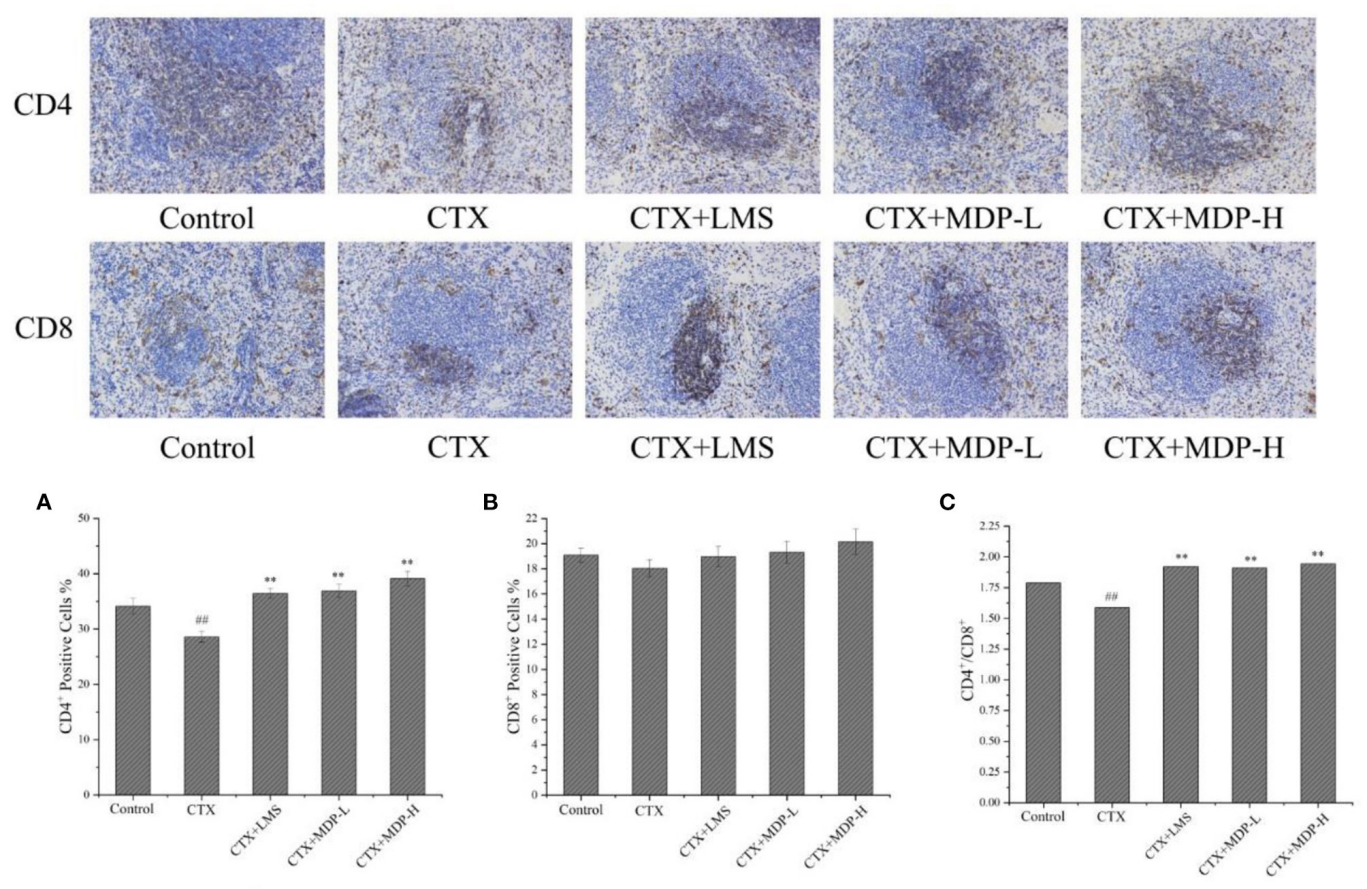
Effect of MDP on the T lymphocyte of spleen. **(A)** Quantitative analysis of the effect of MDP on the CD4^+^ T lymphocyte of spleen. **(B)** Quantitative analysis of the effect of MDP on the CD4+ T lymphocyte of the spleen. **(C)** Effect of MDP on the CD4^+^/CD8^+^ of the spleen. Values are given as means ± S.D (*n* = 3). ^##^*P* < 0.01 vs. control group; **P < 0.01 vs. CTX group.

To investigate the effects of MDP on cellular immunity, CD4^+^ and CD8^+^ T lymphocyte levels were determined by immunohistochemistry. The percentages of splenic CD4^+^ T lymphocytes (Figure 10A) were significantly higher in the MDP-treated groups than in the CTX group. Compared with the CTX group, the increases in CD8^+^ T lymphocytes (Figure 10B) in the MDP-treated groups were not statistically significant. The CD4^+^/CD8^+^ (Figure 10C) of the MDP-treated groups also led to a significant increase when compared with the CTX group, indicating that MDP improved immune function by regulating T lymphocyte subsets.

#### Effect of MDP on the TLR4-MyD88 Pathways *in vivo*

**Figure 11:**
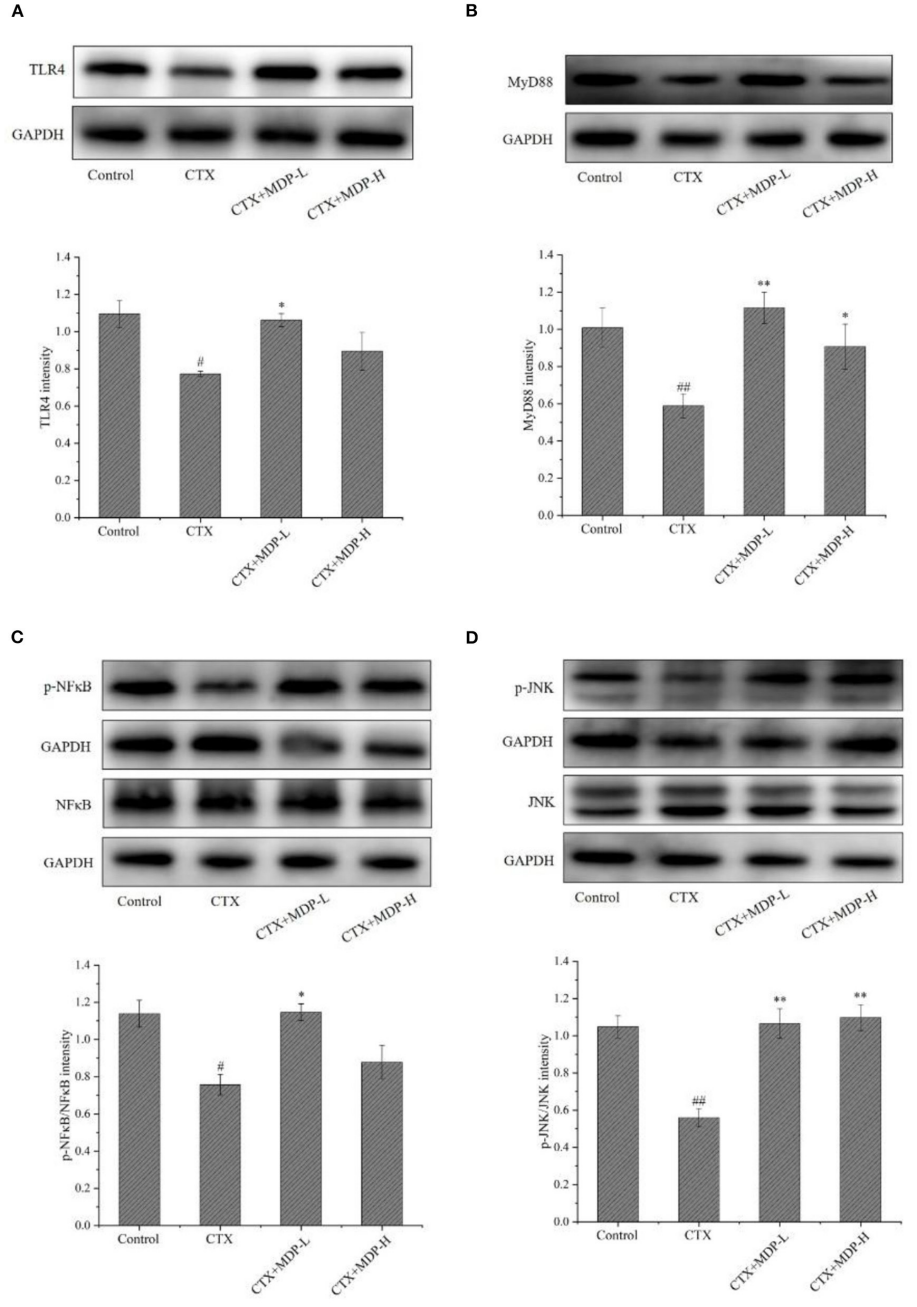
Effect of MDP on the TLR4-MyD88 pathways *in vivo*. **(A)** The protein expressions of TLR4. **(B)** The protein expressions of MyD88. **(C)** The protein expressions of p-NFκB. **(D)** The protein expressions of p-JNK. Values are given as means ± S.D (*n* = 3). ^#^*P* < 0.05, ^##^*P* < 0.01 vs. control group; *P < 0.05, **P < 0.01 vs. CTX group.

**Figure 12:**
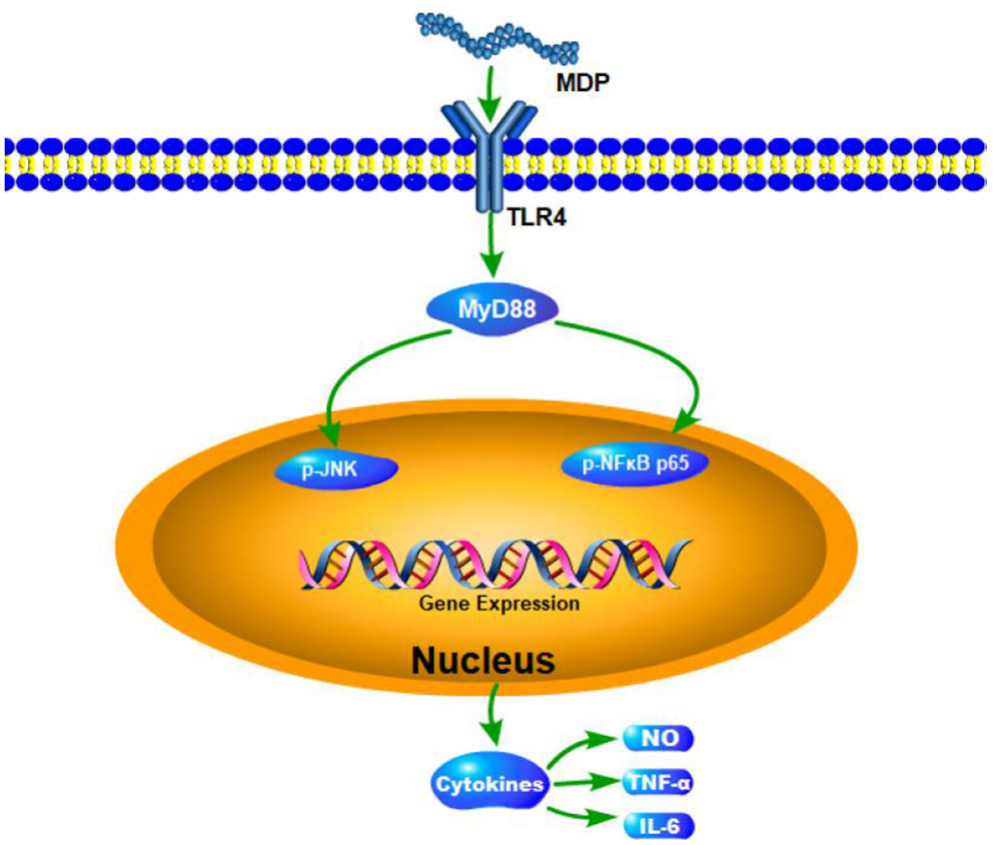
The proposed mechanism underlying the immunological effects of MDP.

TLR4, a canonical receptor for LPS, is known to recognize a variety of natural polysaccharides. TLR4 has two distinct downstream signaling pathways, including MyD88- and TRIF-dependent signaling pathways. Subsequent activation of MAPK/NF-κB signaling pathway by MyD88-dependent pathway induces the secretion of effector cytokines (29). To further verify whether TLR4-MyD88 mediates the immunomodulatory effects of MDP, we measured the effect of MDP on the protein expression levels of key nodes in the TLR4-MyD88 signaling pathway in the CTX-treated mice. The results showed MDP significantly elevated the protein expression (Figure 11) of TLR4, MyD88, p-NFκB, and p-JNK. The results demonstrated that MDP activates MyD88-dependent signaling pathways through TLR4 to promotes immune activity (Figure 12).

## Discussion

According to the findings of the present research, MDP presented significant immunomodulatory activity as a plant-originated polysaccharide. In recent years, more and more glucans with immunomodulatory activity have been reported. Bao et al. (30) found that branched (1 → 3)-α-d-Glc*p* isolated from *Ganoderma lucidum* spores may boost both *in vitro* and *in vivo* lymphocyte proliferation as well as antibody production. Yang et al. (31) extracted a polysaccharide called NGP from ginger that had a main chain of 1,4-linked α-D-Glc*p* and α-D-Glc*p* residues branched at the C-6 position. NGP could boost macrophage proliferation significantly without cytotoxicity and promote immune substances production (NO, TNF-α, IL-1β, and IL-6). Nair et al. (32) revealed that α-glucan containing a (1 → 4) linked backbone and (1 → 6) linked branches from *Tinospora cordifolia* presented distinctive immune-stimulating characteristics. Many polysaccharides containing (1 → 6)-α-D-glucan have great immunological activity. Zhao et al. (33) reported that (1 → 6)-α-D-glucan from the *Ipomoea batatas* root could contribute to an improvement in the immune system and be considered as a biological response modifier. Yang et al. (34) extracted an α-(1 → 6)-D-glucan from banana, its immunostimulatory activities had similarities to a clinical immunostimulatory drug known as β-(1 → 3)-D-glucan. Similarly, in our study, MDP could also activate macrophages to promote immune activity, which may be related to the inclusion ofα-(1 → 6)-D-glucan.

In summary, a new water-soluble polysaccharide called MDP containing a molecular weight of 6.16 × 10^3^ Da was acquired in the present research with the use of DEAE-52 cellulose, Sephacryl S-100, and Sephadex G-50 column chromatography. MDP is an α-D-glucan, which has a (1 → 6)-linked backbone branched at the C-3 position with a glucosyl residue. MDP could remarkably enhance the proliferation, phagocytosis, and release of ROS, NO, TNF-α, and IL-6 factors in RAW264.7 cells. For immunological activity *in vivo*, MDP could significantly increase the thymus and spleen indices, enhance the macrophage function, increase the level of cytokine (IL-6 and TNF-α) and immunoglobulin (IgM) in the serum and regulate T lymphocyte subsets. Furthermore, MDP elevated the expression of the critical nodes in the TLR4-MyD88 signaling pathways *in vivo*. The results indicate that MDP has the capacity of becoming an immunomodulator and could further be applied in the pharmaceutical as well as functional food industries.

## Data Availability Statement

The raw data supporting the conclusions of this article will be made available by the authors, without undue reservation.

## Ethics Statement

The animal study was reviewed and approved by Shandong University of Traditional Chinese Medicine Welfare Ethics Review Committee.

## Author Contributions

PY: conceptualization, methodology, formal analysis, investigation, and writing-original draft. JJ: conceptualization, methodology, formal analysis, investigation, and data curation. FW: resources and validation. YLi: conceptualization and methodology. YM: validation and writing-review and editing. BD: project administration. YZ: supervision, project administration, validation, and visualization. YLiu: supervision, project administration, validation, and writing—review and editing. All authors contributed to the article and approved the submitted version.

## Funding

We received support in the form of grants from the National Natural Science Foundation of China (81973218), Natural Science Foundation of Shandong Province (ZR2019MH082), Taishan Industry Leading Talents Project (tscy20200410), Technology Development Program of TCM of Shandong Province (2019-0024), Open Projects Fund of NMPA Key Laboratory for Quality Research and Evaluation of Carbohydrate-Based Medicine (No. 2021QRECM02), Shandong Key Laboratory of Carbohydrate Chemistry and Glycobiology, Shandong University (2021CCG05), and Scientific Foundation of Shandong University of Traditional Chinese Medicine (2018zk15).

## Conflict of Interest

BD was employed by Sishui Siheyuan Culture and Tourism Development Company, Ltd. The remaining authors declare that the research was conducted in the absence of any commercial or financial relationships that could be construed as a potential conflict of interest.

## Publisher's Note

All claims expressed in this article are solely those of the authors and do not necessarily represent those of their affiliated organizations, or those of the publisher, the editors and the reviewers. Any product that may be evaluated in this article, or claim that may be made by its manufacturer, is not guaranteed or endorsed by the publisher.
