# Supplementary figures and images for: Structure Characterization, Immunological Activity, and Mechanism of a Polysaccharide From the Rhizome of Menispermum dauricum DC
Source: Front Nutr. 2022 Jun 15;9:922569. doi: 10.3389/fnut.2022.922569 (PMC9240474; doi:10.3389/fnut.2022.922569)

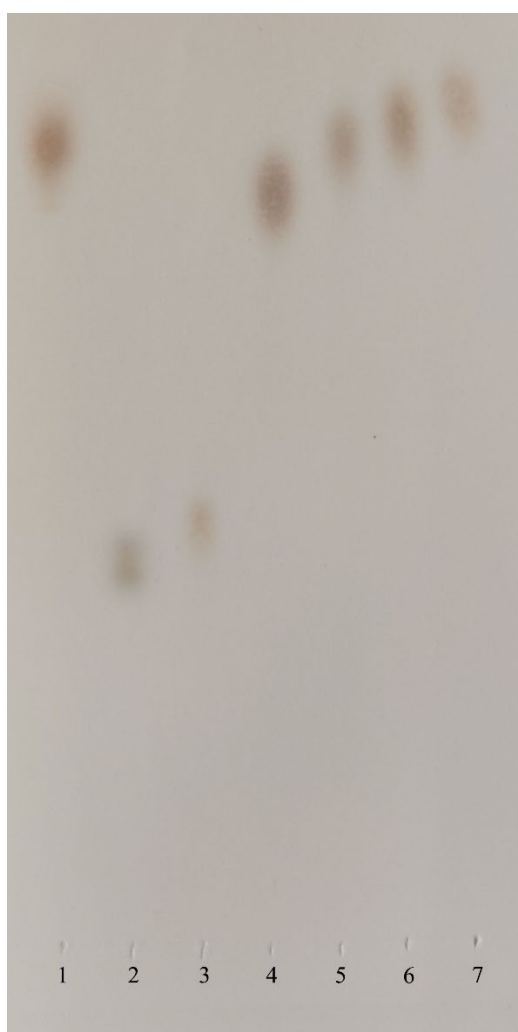

Fig. S1 TLC of MDP. 1. MDP; 2. GalUA; 3. GlcUA; 4. Gal; 5. Glc; 6. Man; 7. Ara;

Supplement: Supplementary file 1 [file Image_1.pdf]
